# Remediation of copper contaminated soil by using different particle sizes of apatite: a field experiment

**DOI:** 10.1186/s40064-016-2492-y

**Published:** 2016-07-26

**Authors:** Jinfeng Xing, Tiantian Hu, Long Cang, Dongmei Zhou

**Affiliations:** 1 Key Laboratory of Soil Environment and Pollution Remediation, Institute of Soil Science, Chinese Academy of Sciences, Nanjing, 210008 China; 2 University of Chinese Academy of Sciences, Beijing, 100049 China

**Keywords:** Apatite, Particle size, Copper, Adsorption, Field experiment

## Abstract

The particle size of apatite is one of the critical factors that influence the adsorption of heavy metals on apatite in the remediation of heavy metal contaminated soils using apatite. However, little research has been done evaluating the impact of different particle sizes of apatite on immobilization remediation of heavy metal polluted soils in field. In this study, the adsorption isothermal experiments of copper on three kinds of apatite was tested, and the field experiment by using different particle sizes apatite [nano-hydroxyapatite (NAP), micro-hydroxyapatite (MAP), ordinary particle apatite (OAP)] at a same dosage of 25.8 t/ha (1.16 %, W/W) was also conducted. Ryegrass was chosen as the test plant. The ryegrass biomass, the copper contents in ryegrass and the copper fractionations in soil were determined after field experiments. Results of adsorption experiments showed that the adsorption amounts of copper on OAP was the lowest among different particles. The adsorption amounts of copper on MAP was higher than NAP at high copper equilibrium concentration (>1 mmol L^−1^), an opposite trend was obtained at low copper concentration (<1 mmol L^−1^). In the field experiment, we found that the application of different apatites could effectively increase the soil pH, decrease the available copper concentration in soil, provide more nutrient phosphate and promote the growth of ryegrass. The ryegrass biomass and the copper accumulation in ryegrass were the highest in MAP among all treatments. The effective order of apatite in phytoremediation of copper contaminated field soil was MAP > NAP > OAP, which was attributed to the high adsorption capacity of copper and the strong releasing of phosphate by MAP.

## Background

Soil contamination is an excess of any compound or element, which results in serious impact on ecosystems, groundwater, agricultural productivity and human health (Adriano 2001). Copper is an essential element: it forms organic complexes and metalloproteins, especially haemoglobin (Kos and Lestan 2004). But high concentration of Cu could cause serious harms to biota and human (Adrees et al. 2015). Therefore, effective amendment and proper treatment to decrease its availability and mobility become necessary.

Apatite is a common name for different minerals with the formula X_5_(YO_4_)_3_ (OH, F, Cl), where X is divalent metal, such as Ca, Ba, Pb, etc., and Y is phosphorous, possible replaced by other elements such as As, V, C (Narasaraju and Phebe 1996). Studies focusing on immobilization of heavy metals by apatite such as natural mineral phosphate and synthetic apatite have been extensively reported (Laperche et al. 1996; Ma et al. 1995; Miretzky and Fernandez-Cirelli 2008). Hydroxyapatite (HAP) is a member of apatite mineral family, with the formula Ca_10_(PO_4_)_6_(OH)_2_, and has a high adsorption capacity for divalent heavy metal ions and has been used for wastewater treatment (Corami et al. 2008; Ma et al. 1994; Sandrine et al. 2007; Smiciklas et al. 2006) and soil remediation (Chaturvedi et al. 2006; Keller et al. 2005). Metal ions such as Pb(II), Cu(II), Co(II), Zn(II), and Cd(II) are successfully immobilized by adsorption and precipitation on the surface of HAP (Corami et al. 2007; Lusvardi et al. 2002; Sandrine et al. 2007; Smiciklas et al. 2006). Moreover, Chen et al. (2006) indicated that the solubility and grain size of rock phosphate could affect the effectiveness of the amendment in in situ remediation technology, and they concluded that the rock phosphate with small grain size (<35 μm) was superior to grain sizes larger than 35 μm at reducing heavy metals uptake in plant. Sugiyama et al. (2002) found that the exchange capacity of apatite of particle size under 0.85 mm to Pb(II) is much better than that of particle size from 0.85 to 1.70 mm. In particular, nano-materials represent a promising application in some areas due to their high surface areas, small sizes and special chemical reaction (Zhang et al. 2010). Wang et al. (2009) found that nanoscale HAP exhibits a strong adsorption for Cu, and is a good adsorbent for immobilization of heavy metals. Liu and Zhao (2007) found nano-particles had better performance than normal particles during studying the immobilization of Cu(II) in soil using iron phosphate.

Although the immobilization of heavy metals using apatite has been proved successfully in the laboratory, the remediation of heavy metal contaminated soils by nano-hydroxyapatite (NAP), micro-hydroxyapatite (MAP) and ordinary particle size of apatite (OAP) in the field is very limited. Thus, the objectives of this study were to evaluate the Cu adsorption capacity on different particle size of apatite (NAP, MAP, OAP) and the remediation ability of Cu contaminated soil by different apatites in the field condition.

## Methods

### Soil

The test site is located in Guixi city, Jiangxi province, China (116°55′E, 28°12′N). This area is influenced by Southeast Asia Monsoon, so the climate is warm and humid. The soil was polluted by the sewage and waste gas from Cu smelting factory. The test field has been abandoned for many years and there are many serious phenomena such as desertification, acidification and heavy mental pollution. The soil pH, organic matter content, available N, P and K concentration were 4.25, 30.8 g kg^−1^, 46.7, 122 and 41.5 mg kg^−1^, respectively. The total Cu, Zn, Pb and Cd concentrations were 580, 85.5, 37.6 and 0.35 mg kg^−1^, respectively.

### Materials

**Fig. 1:**
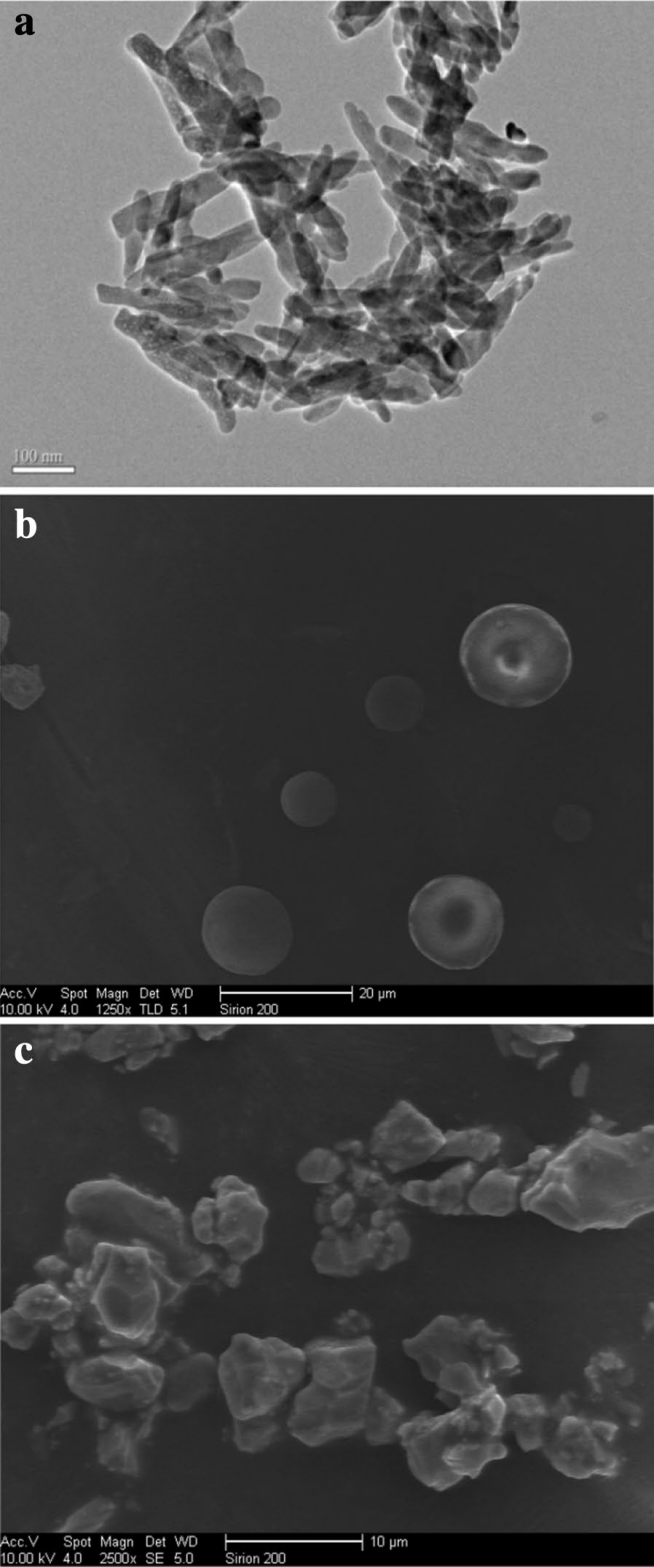
The TEM images of different apatites. **a** nano-hydroxyapatite (NAP), **b** micro-hydroxyapatite (MAP), **c** ordinary particle size of apatite (OAP)

Nano-hydroxyapatite (NAP), micro-hydroxyapatite (MAP) and ordinary particle size of apatite (OAP) were used as adsorption materials and purchased from Nanjing emperor nano material Co. Ltd. and Xintai phosphorus chemical plant. The basic physicochemical properties and the TEM imaging of apatites are shown in Table 1 and Fig. 1. Ryegrass (*Lolium perenne* L.), supplied by Nanjing Shenzhou Seeds Industry Co., Ltd., was chose as the test plant.

**Table 1:** The basic properties of the different apatites

Material	Particle	Purity (%)	Shape	BET (m^2^ g^−1^)	pH	Zeta potential (mV)	Pb (mg kg^−1^)	Cu (mg kg^−1^)	Zn (mg kg^−1^)	Cd (mg kg^−1^)	Ca/P
NAP	60 nm	>96	Acicular	68.8	7.07	−5.55	10.8	3.77	9.81	0.474	1.70
MAP	12 μm	>96	Sphere	42.8	7.23	−44.4	8.94	21.6	20.5	0.454	1.61
OAP	0.15 mm	>96	Bulk	1.25	9.70	−17.0	18.2	11.2	18.6	0.469	2.12

### Adsorption isothermal experiments

The adsorption isothermal experiments were carried out in 50 mL polypropylene centrifugation tubes by mixing 0.05 g of different apatites with 25 mL 0.01 mol L^−1^ NaNO_3_ containing 25 mmol L^−1^ MES (4-Morpholineethanesulfonic acid hydrate). The pH of suspensions were adjusted to 5.5 by diluted HCl or NaOH. Afterwards, 5 mL of 0.01 mol L^−1^ NaNO_3_ with different concentration of Cu(NO_3_)_2_ were added into the tubes. The initial concentration of copper in the solutions were 0, 0.2, 0.4, 1.0, 1.6, 2.0, 5.0 and 10.0 mmol L^−1^, respectively, and each treatment was duplicated. All samples were shaken for 24 h at 25 °C, followed by centrifuging at 9000 rpm for 15 min. The supernatants were filtered through a 0.22 μm Millipore filter. The Cu concentrations in the filtrate were measured by atomic absorption spectroscopy (AAS). Adsorbed copper was quantified by the difference between initial and final copper concentrations in solution after control correction. The adsorption isotherms were fitted by the *Langmuir* and *Freundlich* equations (Eqs. 1 and 2, respectively).

1 $${\text{Q}}_{\text{e}} = {\text{Q}}_{\text{m}} {\text{K}}_{\text{L}} {\text{C}}_{\text{e}} /\left( {1 + {\text{K}}_{\text{L}} {\text{C}}_{\text{e}} } \right)$$

2 $${\text{Q}}_{\text{e}} = {\text{K}}_{\text{f}} {\text{Cn e}}$$

where C_e_ (mmol L^−1^) is the equilibrium concentration of copper in solution, Q_m_ (mmol kg^−1^) is the adsorbed quantity of copper, Q_m_ (mmol kg^−1^) and K_L_ (L kg^−1^) indicate the maximal monolayer adsorption capacity and a constant related to adsorption energy, respectively. K_f_ and n are Freundlich constants giving an estimation of the adsorption capacity and intensity, respectively.

### Field experiment

The field plot trials were conducted to investigate the remediation effect by using different particle size of apatite in the Cu contaminated field. Triplicated trial plots (2 m × 2 m) were laid out by a randomized block design. The trial plots were separated by 0.5 m high PVC sheets with 0.2 m above ground and 0.3 m underground. The application dosages of the apatite were 1.16 % (W/W) of the topsoil (0–20 cm) weight (25.8 ton ha^−1^). The treatments were labeled as NAP, MAP, OAP and CK (without any amendment), respectively.

On November 13, 2010, the site was ploughed and harrowed. In order to avoid the suspension and agglomeration of apatites (especially NAP), the apatite powders were spread on the soil surface and mixed evenly with topsoil, then each plot was irrigated with 100 L of tap water. In each plot, 20 g of ryegrass seeds was sown and 0.5 kg of compound fertilizers (the total content of N, P_2_O_5_ and K_2_O were 15 %) was applied. After 18 weeks (the first harvest, March 29, 2011) and 21 weeks (the second harvest, April 22, 2011) growing, the aboveground biomass of ryegrass was harvested, and then 0.04 kg of the urea was applied per plot. After 23 weeks of growth (the third harvest, May 8, 2011), the shoot and root of ryegrass were harvested, and the soil samples were also collected. The shoot samples was thoroughly washed by tap water and rinsed by deionized water. The root samples were thoroughly washed using a 2-step washing progress, which consisted of a first wash with EDTA (20 mmol L^−1^) and a second rinse with deionized water. Both shoot and root samples were preliminarily dried at 105 °C for 30 min, and then switched to 70 °C until a constant weight has been reached (about 48 h). The dry matters were grounded to fine powders using a mechanical grinder after the yields were recorded. The soil samples were air dried and sieved through 2 and 0.149 mm sieves before measurements.

### Analytical methods

The physical structures of different apatites were imaged by Transmission Electron Microscope (TEM, JEOL TEM-2100,Japan). The mineral phases were analyzed using X-ray Diffraction Spectrometer (XRD, Rigaku Ultima IV, Japan) with the diffraction pattern compared to the referenced XRD standard map (Joint Committee on Powder Diffraction and Standards, NO 09-0432). The FTIR spectra was record on a Nicolet 380 patterns (USA) of the solid were taken (scan in 400–4000 cm^−1^), as about 2 mg samples were suspended in about 150 mg of KBr. Zeta potential was determined by NanoBrook 90Plus PLAS (Brookhaven Instruments, America). The specific surface area was measured by the Brunauer-Emmett-Teller (BET) method using the specific surface area automatic analyzer (Quantachrome Autosorb-iQ, America). The pH of apatites were measured in suspension with the carbon dioxide-free distilled water to solid ratio of 1:6 (W/V) by using pH meter (pHS-2B, Shanghai Rex Electric Chemical Co. Ltd., China). The apatites were digested using HNO_3_-HF-HClO_4_ (10:10:1, V/V) mixture and the digestion solutions were analyzed to quantify the concentrations of Cu, Zn, Pb, Cd, Ca and P in apatites using a flame atomic absorption spectrophotometer (Hitachi Z-2000, Japan).

Soil pH, organic matter content and available phosphorus contents were analyzed based on the method given by Lu (2000). The soil pH were measured in suspension with the carbon dioxide-free distilled water to soil ratio of 2.5 (V/W) by pH meter (pHS-2B). Soil organic matter content was analyzed by dichromate oxidation method. Available soil phosphorus was extracted by the mixed solution contained 0.03 mol L^−1^ NH_4_F and 0.025 mol L^−1^ HCl and determined by ICP (PerkinElmer Optima 8000).

To determine the total concentration of metals in soil, the soil samples were passed through 0.149 mm pore-size sieve and dried at 105 °C for 4 h, and then digested in an HNO_3_-HF-HClO_4_ (10:10:1, V/V) mixture. Blank and soil standard materials (GBW-07401 and GBW-07406) (China National Center for Standard Material) were used for quality control. The Cu fractions were identified by the BCR sequential extraction method from the European Community Bureau of Reference (Rauret et al. 2000), and the acid soluble, reducible, oxidizable, and reducible fractions of Cu were extracted by 0.11 mol L^−1^ acetic acid, 0.1 mol L^−1^ hydroxylamine hydrochloride (pH 2), 30 % W/V H_2_O_2_ and 1 mol L^−1^ NH_4_OAc (pH 2), HNO_3_–HF–HClO_4_ (1:2:1, V/V), respectively. The recovery of total Cu concentration was controlled in 90–110 %. TCLP (Toxicity Characteristic Leaching Procedure) method was used to estimate the variation of Cu toxicity in amended soils. This procedure diluted a 5.7 mL glacial acetic acid in 1 L deionized water and used 20 mL of this solution (pH 2.88) to extract copper from the 1 g of soil samples (Sun et al. 2006).

For the Cu concentration in ryegrass shoots and roots, about 0.5 g sample was weighed in a 50 mL triangular flask with mixture of HNO_3_ (20 mL) and H_2_O_2_ (4 mL) and left at room temperature overnight. Then, the samples were digested on an electric heating plate (120–180 °C). Blank and standard reference materials (GBW-10015 and GBW-10010) (spinach leaves and rice grain, China National Center for Standard Material) were used for quality control in the digestion and analysis processes. The Cu concentrations of in digestion solutions were analyzed using a flame atomic absorption spectrophotometer (Hitachi Z-2000, Japan).

### Statistical analysis

SPSS 18.0 and Excel 2003 were used for statistical analysis. *Langmuir* and *Freundlich* equations were applied to fit the copper adsorption isothermals. The results of field experiments were subjected to analysis by one-way analysis of variance (ANOVA) followed by Duncan multiple comparisons (*p* < 0.5) using SPSS 18.0.

## Results and discussion

### Characteristics of different apatites

**Fig. 2:**
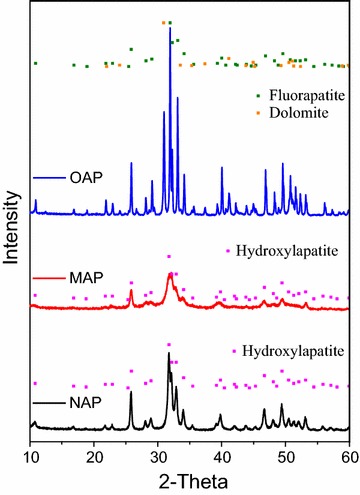
X-ray diffraction (XRD) pattern of different apatites

**Fig. 3:**
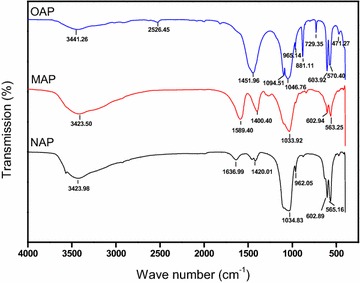
Fourier transform infrared spectroscopy (FTIR) spectra of different apatites

TEM images (Fig. 1) showed that the shapes of NAP, MAP and OAP were acicular, sphere and bulk, respectively. The order of particle size of different apatite was NAP < MAP < OAP, which was on the opposite trend with the specific surface area by the BET method. The results of XRD analysis showed NAP and MAP consisted with the standard card of hydroxyapatite (JCPDS 9-432), and fluorapatite (65 %) was found as the primarily mineral in OAP, and the second most abundant mineral was dolomite (30 %), followed by hydromica (2 %), talcum (2 %) and kaolinite (1 %) (Fig. 2). The XRD and FTIR spectrograms (Figs. 2, 3) indicated the OAP is not pure apatite. Some other minerals (dolomite, hydromica, etc.) didn’t contain phosphorus, which decreased the phosphorus content in OAP and increased the Ca/P ratio of OAP to 2.12. The Ca/P molar ratio of NAP and MAP were 1.70 and 1.61, respectively, which is close to the ideal ratio of 1.67.

Zeta potential of MAP was −44.4 mV and higher than NAP and OAP (absolute value), which indicated that MAP preferentially disperses in soil and mixes with soil components compare to NAP and OAP. The concentrations of heavy metal in apatite were 3.77–21.6 mg kg^−1^ for Cu, 9.81–20.5 mg kg^−1^ for Zn, 8.94–18.2 mg kg^−1^ for Pb and 0.454–0.474 mg kg^−1^ for Cd, respectively, and slightly higher than the concentration previously reported (Cui et al. 2013, 2014).

### Adsorption of Cu on different apatites

**Fig. 4:**
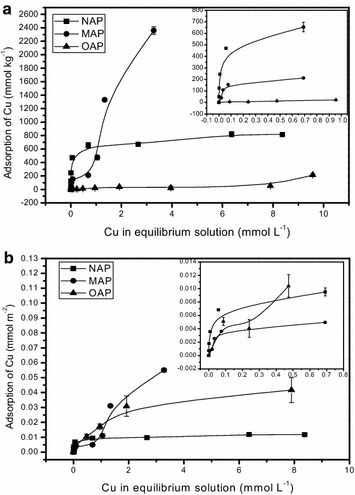
Adsorption isotherms of Cu on the three kinds of apatite at pH 5.5 (The unit of Cu absorption amount in  y-axis were expressed by mmol kg^-1^ (**a**) and mmol m^-2^ (**b**), respectively)

Adsorption isotherms of Cu on apatites at pH 5.5 are given in Fig. 4. The adsorption of Cu on apatite gradually increased with the increase of copper concentration in the equilibrium solution, which is in agreement with those reported by Wang et al. (2009) and Cui et al. (2014). Figure 4a show the adsorption of Cu in mmol kg^−1^. In case of lower Cu concentration (below 1.0 mmol L^−1^) in equilibrium solution, the adsorption trend (from high to low capacity) of apatites was NAP > MAP > OAP. The adsorption trend was similar to the study given by Ma et al. (1995), in which it has been suggested that for phosphate rock, higher specific surface area increased its dissolution rate and promoted the adsorption and immobilization of heavy metals. The adsorptions of Cu were showed in mmol m^−2^ in Fig. 4b. The Cu adsorption on NAP and MAP were similar with the result in Fig. 4a. That suggested that NAP displayed higher immobilizing effect than MAP due to its high surface area (Table 1). However, there was different between Fig. 4a, b for the Cu adsorption capacity on OAP, which indicated the composition and purity of OAP affected the Cu adsorption on OAP.

However, an interesting phenomenon was observed that the Cu adsorption capacity on MAP increased rapidly and was higher than that on NAP when the equilibrium concentration of Cu exceeded 1.0 mmol L^−1^ (Fig. 4a). The highest Cu adsorption capacity on NAP and MAP were 800, 2300 mmol kg^−1^, respectively, and the adsorption capacity on OAP kept staying at a very low level (below 214 mmol kg^−1^). The parameters for *Langmuir* and *Freundlich* fitting are listed in Table 2, the adsorption isotherms of MAP and OAP in 0–10 mmol L^−1^ were not fitted by *Langmuir* and *Freundlich* models and OAP in 0–1 mmol L^−1^ was not fitted by *Langmuir* model. Cheung et al. (2002) found that *Langmuir* equation can used to describe the sorption of Cu by bone char (500–710 μm particle size) mainly composed with hydroxyapatite. This difference might be due to that the different composition and property of sorbent and experiment conditions. The adsorption isotherms of NAP were well fitted by *Langmuir* and *Freundlich* models, and the correlation coefficient (R^2^) were 0.96 (0–10 mmol L^−1^) and 0.98 (0–1 mmol L^−1^) in *Langmuir* models and 0.95 (0–10 mmol L^−1^) and 0.94 (0–1 mmol L^−1^) in *Freundlich* models, respectively. These results showed that the *Langmuir* models is better in describing the Cu adsorption behavior on NAP and the adsorption behavior was mainly a monomolecular adsorption process, similar to studies given by Chen et al. (2010) and Wang et al. (2009). Adsorption reaction of Cu on MAP is divided into two phases (Fig. 2): first, the sorption of Cu on MAP corresponds basically to *Langmuir* equation at low Cu equilibrium concentration (<1.0 mmol L^−1^); second, the adsorption became linear with both rate and capacity increased linearly at high Cu equilibrium concentration (>1.0 mmol L^−1^). These indicated that the adsorption of Cu on MAP might be a multilayer adsorption and the surface of MAP had a strong affinity of Cu at higher Cu concentration. The best metal sorption on apatites need poor crystallinity (Chen et al. 1997a, b). XRD analysis showed the crystallinity of MAP was the poorest, so the Cu adsorption capacity on MAP was the best.

**Table 2:** Langmuir and Freundlich model parameters of different apatite at pH 5.5

Ce (mmol L^−1^)	Material	Langmuir				Freundlich			
		K_L_	Q_m_	R^2^	P	K_F_	1/n	R^2^	P
0–10	NAP	43.15	742.9	0.9628	<0.05	592.1	0.1751	0.9500	<0.01
0–1	NAP	72.73	637.7	0.9815	<0.05	752.9	0.2439	0.9480	<0.01
0–1	MAP	20.84	232.4	0.9616	<0.05	243.6	0.2739	0.8882	<0.05
0–1	OAP	–	–	–	–	22.03	0.7455	0.9460	<0.05

The adsorption isotherms of MAP and OAP in 0–10 mmol L^−1^ were not fitted by Langmuir and Freundlich models and OAP in 0–1 mmol L^−1^ were not fitted by Langmuir models, so their model parameters are not listed

### Effect of different apatites on soil pH, TCLP extracted Cu concentrations

**Fig. 5:**
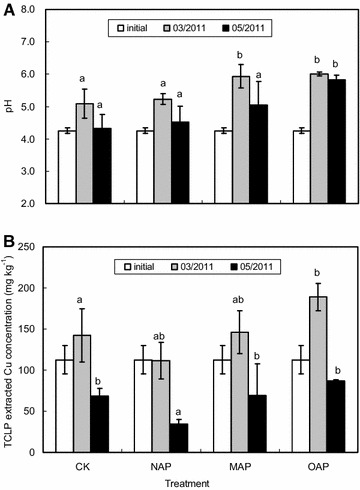
Effect of different apatites on soil pH (**A**) and TCLP extracted Cu concentrations (**B**) (*different letters* indicated significant differences between treatments at the same time (*p* < 0.05)

The addition of NAP, MAP and OAP led to the increase of soil pH (Fig. 5A), which is similar with previous reports (Chlopecka and Adriano 1996; Ma et al. 1995) and probably due to the hydroxyl, calcium and some other basic groups of apatite (Boisson et al. 1999; Knox et al. 2003). However, the soil pH of different treatments significantly increased in March 2011, and then a decrease of soil pH was observed in May 2011. Similarly, Cui et al. (2014) found that with the application of apatite, soil pH significantly increased after 1 year and decreased after 4 years. They speculated that it might be attributed to the fact that test site was located in an acid deposition zone. As shown in Fig. 5A, the soil pH followed the same order of OAP > MAP > NAP > CK in March 2011 and May 2011, which was consisted with the pH order of apatites (OAP > MAP > NAP). Moreover, the soil pH treated by NAP slightly changed whereas significant pH increases were identified in soil treated by OAP and MAP. This indicated that OAP and MAP is more effective in increasing soil pH than NAP under the identical condition.

Figure 5B shows the concentration changes of TCLP extracted Cu caused by the addition of NAP, MAP and OAP, and the concentration increased in March 2011 compared to November 2010. Possibly due to the open experimental system in field, the immobilization process of Cu was relatively slow. But after two months since March 2011, TCLP extracted Cu decreased significantly alone with the order of OAP > CK > MAP > NAP. The results indicated that NAP and MAP were able to immobilize TCLP extracted Cu and NAP was more effective than MAP. However the soil pH in MAP treatment were higher than those in NAP treatment, especially there was significant differences between NAP and MAP in March 2011 (Fig. 5A). The immobilization of Cu by HAP was possibly more dependent on the high adsorption quantity of Cu rather than the soil pH comparing to that by MAP. Nevertheless, more experiments are still needed to further study the effect of soil pH on the immobilization mechanism of Cu by apatite.

### Effect of different apatites on the Cu speciation in soil

The mobility of heavy metals, their bioavailability and related eco-toxicity to plant, depend strongly on their specific chemical fractions (Chen et al. 2014; Wang et al. 2012). Application of amendment can change the chemical fractions of heavy metals in soil and reduce their toxicity (Mackie et al. 2015; Montenegro et al. 2015; Rodriguez-Vila et al. 2015).

The concentration of Cu and the percentages of four fractions in soil estimated by BCR method are shown in Table 3. In the untreated soil (CK), the acid soluble fraction (250 mg/kg, 47.7 %) was predominant followed by reducible fraction (134 mg/kg, 25.5 %) and oxidizable fraction (109 mg/kg, 20.7 %), the lowest percentage was found in residual fraction (31.9 mg/kg, 6.08 %). After application of NAP, MAP and OAP, the Cu concentration in acid soluble fraction decreased significantly from 47.7 % (CK) to 31.2 % (NAP), 39.6 % (MAP) and 40.5 % (OAP), respectively. By contrast, the Cu concentration in reducible fraction increased significantly with the highest increasing in the NAP treatment and the lowest increasing in the OAP treatment. Only small changes in residual fraction were identified among all treatments. The results indicated that the application of apatites reduced the availability of Cu and attenuated its risk by decreasing the high toxic and mobile acid soluble fraction and meanwhile immobilizing Cu to relatively stable reducible and oxidizable fraction. The order of immobilizing is: NAP > MAP > OAP, but there have no significant difference in the acid soluble fraction between NAP and MAP. A previous study (Cui et al. 2013) showed that MAP was more effective than NAP in immobilizing Cu under the greenhouse condition. The difference might be due to the physic-chemical properties of apatites and experiment conditions (pot or field experiment). The particle size of NAP (40 nm) and MAP (3 μm) in Cui’s article (Cui et al. 2013) is smaller than that in our study (60 nm NAP and 12 μm MAP), which could cause a bad dispersibility for NAP in soil and subsequently weak the immobilization effect. Nevertheless, more studies are needed to verify the different immobilization mechanisms of Cu by NAP and MAP under different conditions.

**Table 3:** Effect of different apatites on the Cu fractionations in soil

Treatment	F1		F2		F3		F4		Recovery %
	mg kg^−1^	%	mg kg^−1^	%	mg kg^−1^	%	mg kg^−1^	%	
CK	250 a	47.7	134 c	25.5	109 c	20.7	31.9 a	6.08	93.7
NAP	195 a	31.2	218 a	34.8	180 a	28.6	33.5 a	5.34	110
MAP	234 a	39.6	164 bc	27.8	152 bc	25.7	41.0 a	6.92	106
OAP	244 a	40.5	155 bc	25.7	173 ab	28.7	30.0 a	4.99	99.1

F1, acid soluble fraction; F2, reducible fraction; F3, oxidizable fraction; F4, residual fraction; Mean values followed by different lowercase letters in each column means significantly different (*p* < 0.05, n = 3)

Possible mechanisms of metal retention by apatite included: (1) ion exchange at the surface of apatite (Xu et al. 1994); (2) surface complexation (Cao et al. 2004); (3) precipitation of some amorphous to poorly crystalline, mixed metal phosphates; and (4) substitution of Ca in apatite by other metals during recrystallization (or coprecipitation) (Chen et al. 1997a, b; Xu et al. 1994). According to the report of Lindsay (2001), the structure of hydroxyapatite is similar to that of fluorapatite, F^−^ occupying the OH^−^ sites on the sixfold axis. But hydroxyapatite (log K = 14.46) has a higher solubility than fluorapatite (log K = −0.21). Ma et al. (1995) attributed that the higher Pb removal efficiency to higher solubility and purity of hydroxyapatite than phosphate rocks. Drouet (2015) indicated that Gibbs free energy also estimated the solubility of apatites. Moreover, Kaludjerovic-Radoicica and Raicevicb (2010) suggested that the ΔG^0^ of HAP was greater than that for LA (fluorapatite), and HAP showed a larger affinity for Pb removal by the greater value of the sorption capacity. In this study, the immobilizing capacity of Cu followed the order of NAP > MAP > OAP, and the OAP was primarily fluorapatite and not pure. Therefore, one possibility reason could be the higher solubility of MAP and NAP than OAP. Furthermore, larger specific surface areas were determined for NAP and MAP than OAP (Table 1), which is likely enhancing the sorption of metals and leading to the decrease of the bioavailability of metal (Chen et al. 2006; Zhang et al. 2010). Thermodynamic properties might be different somewhat depending on the grain size of the apatite (Drouet 2015), therefore, more studies about thermodynamic of NAP and MAP need to conduct.

### Effect of different apatites on ryegrass biomass

Table 4 shows the shoot and root biomass of ryegrass in different treatments. Ryegrass was not able to grow in the CK treatment (without the addition of amendment), but application apatites resulted in improved the growth of ryegrass.

**Table 4:** Effect of different apatites on the biomass of ryegrass

Treatment	Biomass of shoot (g)			Biomass of root (g)	Total biomass of shoot (g)	Total biomass of ryegrass (g)
	1st cutting	2nd cutting	3rd cutting			
NAP	314.5 b	670.7 b	343.1 b	232. 9 b	1328 b	1561 b
MAP	2808 a	1819 a	1075 a	501.3 a	5703 a	6205 a
OAP	356.3 b	685.0 c	240.6 b	159.2 bc	1282 b	1441 b

Mean values followed by different lowercase letters in each column means significantly different (*p* < 0.05, n = 3)

**Fig. 6:**
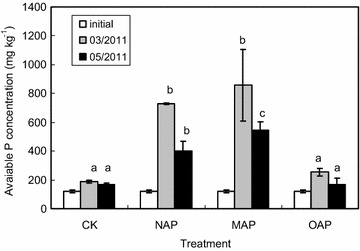
Effect of different apatites on available phosphorus concentration [*different letters* indicated significant differences between treatments at the same time (*p* < 0.05)]

In general, the best growth responses (shoot and root) were observed at MAP addition among all treatments. And there were significant differences between this treatment and others. The trend of shoot, root and total biomass of ryegrass was MAP > NAP > OAP, although there was no significant difference between NAP and OAP. It is well known that nutrient availability is of primary importance to the productivity. It can be seen from Fig. 6 that the apatites significantly increased the concentration of available phosphorus and the increasing order was MAP > NAP > OAP, especially there have significant difference between MAP and NAP in May 2011. Therefore, the release of phosphorus by these different apatites was one of the important factors to the biomass variation. The Cu adsorption on NAP was higher than that on OAP (Fig. 4), but no significant difference of ryegrass biomass was found between NAP and OAP. So another important factor of ryegrass growth was the soil pH, 5.83–6.01 in the OAP treatment, significantly higher than the soil pH (4.53–5.23) in the NAP treatment (Fig. 5). The neutral soil pH was beneficial to the growth of ryegrass (Mora et al. 2005; Rosas et al. 2007).

### Effect of different apatites on the Cu content and accumulation in ryegrass

Table 5 shows the Cu content and accumulation in ryegrass of different treatments. After applying apatites with different particle sizes, the Cu contents in the shoot of different cutting time was different. The Cu contents in shoot after the first cutting was much higher than those of the second and the third cutting excepted for the NAP treatment, which may be related to the dilution effect resulting from the significantly increasing of ryegrass biomass in the second and the third cutting. There was no significant difference of Cu contents in the shoot among different treatments, except that the Cu content in the third cutting shoot of the MAP treatment was significantly less than those in other treatments. However, the Cu content of root in the MAP was the highest in all treatments, and was about 1.69, 3.90 times that in NAP, OAP, respectively. According to the report of Laperche et al. (1997), the treatments with high quantities of phosphorus added resulted in the content of Pb in the plant roots the same or higher than the untreated soil. Therefore, we speculated that the higher Cu content of root in the MAP may be attributed to its highest phosphorus concentration (Fig. 6).

**Table 5:** Effect of different apatites on Cu concentration and Cu accumulation in ryegrass

Treatment	Cu concentration (mg kg^−1^)				Cu accumulation (mg plot^−1^)	
	Shoot			Root	Shoot	Root
	1st cutting	2nd cutting	3rd cutting			
NAP	182.3 a	91.6 a	124.3 a	7373 ab	221.4 b	1952 ab
MAP	249.3 a	154.6 a	63.20 b	12449 a	1143 a	5580 a
OAP	235.7 a	178.9 a	128.1 a	3190 b	206.1 b	518.4 b

Mean values followed by different lowercase letters in each column means significantly different (*p* < 0.05, n = 3)

The Cu accumulation amount in shoot (1143 mg plot^−1^) and root (5580 mg plot^−1^) in the MAP treatment were 5.16 and 2.86 times higher than that in the NAP treatment, from which it was concluded that the ryegrass combined MAP was more effective than NAP or OAP in removing Cu from soil. The overall trend of Cu accumulation was MAP > NAP > OAP, which was corresponding to the ryegrass biomass. This field study indicated that the ryegrass biomass was the major factor for Cu accumulation, whereas the different particle size of apatite had small effect on the Cu content in the shoot of ryegrass.

## Conclusions

Our study clearly showed that apatites effectively increased the soil pH and improved the growth of ryegrass, decreased the concentration of available Cu in soil, and promoted the Cu transformation from active to stable fraction in field experiment. Although the specific surface area of NAP was larger than MAP and OAP, the adsorption of Cu on MAP was better than that on NAP in high Cu concentration (>1 mmol L^−1^) in adsorption experiment. The ryegrass biomass and the copper accumulation in ryegrass were the highest in MAP among all treatments. The effective order of apatite in phytoremediation of copper contaminated field soil was MAP > NAP > OAP, which was attributed to the high adsorption capacity of copper and the stronger releasing of phosphate by MAP.
